# Predicting the impact of climate change on the suitable habitat of *Populus qiongdaoensis* in Hainan Island using MaxEnt modeling

**DOI:** 10.1038/s41598-026-36841-3

**Published:** 2026-04-29

**Authors:** Yang Tian, Tong Zhang, Qinuo Li, Baochang Cheng, Ruobing Wei, Yong Zeng, Jingkai Zhang, Jianguo Zhang, Zhaoshan Wang

**Affiliations:** 1 Key Laboratory of Silviculture of the State Forestry Administration, Research Institute of Forestry, Chinese Academy of Forestry, Beijing, 100091 China; 2 Powerchina Northwest Engineering Corporation Limited, Xi’an, 710100 China; 3 China Three Gorges University, Yichang, 443002 China

**Keywords:** *Populus qiongdaoensis*, MaxEnt, Suitable habitat, Environmental factors, Climate change, Ecology, Ecology, Environmental sciences

## Abstract

*Populus qiongdaoensis* is a rare and endemic tree species exclusively located in Hainan Island, China, holding important value for species conservation and ecosystem function. However, its natural populations are threatened by habitat fragmentation and climate change. Based on 42 occurrence records and environmental data, we employed the MaxEnt model to predict the species’ suitable habitats, analyze the dominant environmental factors influencing its distribution, and assess changes under future climate scenarios (using the BCC-CSM2-MR model for the 2050s and 2090s under four SSP pathways). The results showed that elevation, mean temperature of the driest quarter (bio9), and precipitation seasonality (bio15) were the most crucial factors affecting its distribution. Currently, the suitable habitats are primarily located in the Bawangling and Wuzhishan mountain regions, covering approximately 9.6% of the island’s area. Under future scenarios, the total suitable habitat area is projected to increase by 7.4% to 34.2% across most projections. However, the area of highly suitable habitat is predicted to decrease notably under the low-emission scenario (SSP126) in the 2050s. Furthermore, the centroid of the species’ distribution is expected to shift northeastward over time, though remaining within the Wuzhishan region. This study delineates priority conservation areas and provides a critical scientific basis for the habitat protection, population restoration, and sustainable management of *P. qiongdaoensis* under climate change.

## Introduction

 Climate change poses a major threat to global biodiversity. Tropical regions and island ecosystems are especially vulnerable, due to their high levels of endemism and the restricted ranges of many species^1,2^. In recent years, understanding how climate change affects plant geographical distributions has become a key research focus in plant ecology and biogeography^3–5^. Global temperatures are projected to rise by 1.5 °C this century^6^. Such changes can reduce a species’ adaptation to local environmental conditions, often leading to shifts in its distribution, population size, and genetic diversity patterns^7,8^. Therefore, predicting potential suitable habitats, identifying the key ecological factors that limit distributions, and modelling range shift pathways are crucial for future biodiversity conservation and strategy planning^9,10^.

Island ecosystems are especially sensitive to these changes because of their limited spatial extent, restricted dispersal opportunities, and high endemicity^11,12^. Hainan Island, a typical tropical island system in southern China, has experienced pronounced climatic changes in recent decades, including rising temperatures, increasing precipitation variability, intensified typhoon activity, and gradual sea-level rise^11,13–15^. These environmental shifts are likely to substantially alter habitat suitability for endemic plant species, particularly tree species with narrow ecological niches and restricted distributions.

Species distribution models (SDMs) are widely used to estimate potential suitable habitats by relating species occurrence records to environmental variables^16,17^. Among them, the Maximum Entropy (MaxEnt) model has been extensively applied due to its strong predictive performance using presence-only data and its suitability for species with limited occurrence records^18–20^. SDMs have proven particularly valuable for assessing the impacts of climate change on plant species and identifying areas of conservation priority^4,21,22^. However, most existing studies focus on temperate or widely distributed taxa, while endemic tropical tree species, especially those confined to island ecosystems, remain comparatively understudied.

*Populus qiongdaoensis* (Salicaceae) is a tree species endemic to Hainan Island, China, holding notable ecological importance as a component of island forests and possesses significant genetic value due to its unique evolutionary lineage^23^, and is considered as endangered species according to the 7th criteria for established by the International Union for the Conservation of Nature and Natural Resources^23^. As the only *Populus* species adapted to tropical climates, it represents a unique lineage for studying tropical adaptation and island forest dynamics^23,24^. Genomic studies have highlighted its distinct evolutionary history, yet its natural populations are threatened by habitat fragmentation and climate change. Assessing its habitat suitability is thus urgent for conservation planning. Currently, the potential effects of climate change on *P. qiongdaoensis* remain unknown. Given its tropical affinity, factors such as precipitation seasonality, extreme weather events, and topographic constraints may be more critical than temperature alone in limiting its distribution^25,26^. We hypothesize that future climate change may further fragment and reduce suitable habitats due to increasing climatic variability and extreme events on Hainan Island.

In this study, we used the MaxEnt model to (1) identify the key climatic factors shaping the distribution of *P. qiongdaoensis*, (2) map its current potential suitable habitats on Hainan Island, and (3) project changes in habitat suitability under future climate scenarios. By focusing on a tropical endemic *Populus* species confined to an island ecosystem, this study addresses an important knowledge gap in species distribution modeling under climate change. Our results provide valuable insights for conservation planning, including the identification of priority areas for in situ and ex situ conservation, and contribute to a broader understanding of how tropical tree species may respond to ongoing and future climatic changes.

## Results

### Evaluation of model optimization results and accuracy

**Fig. 1:**
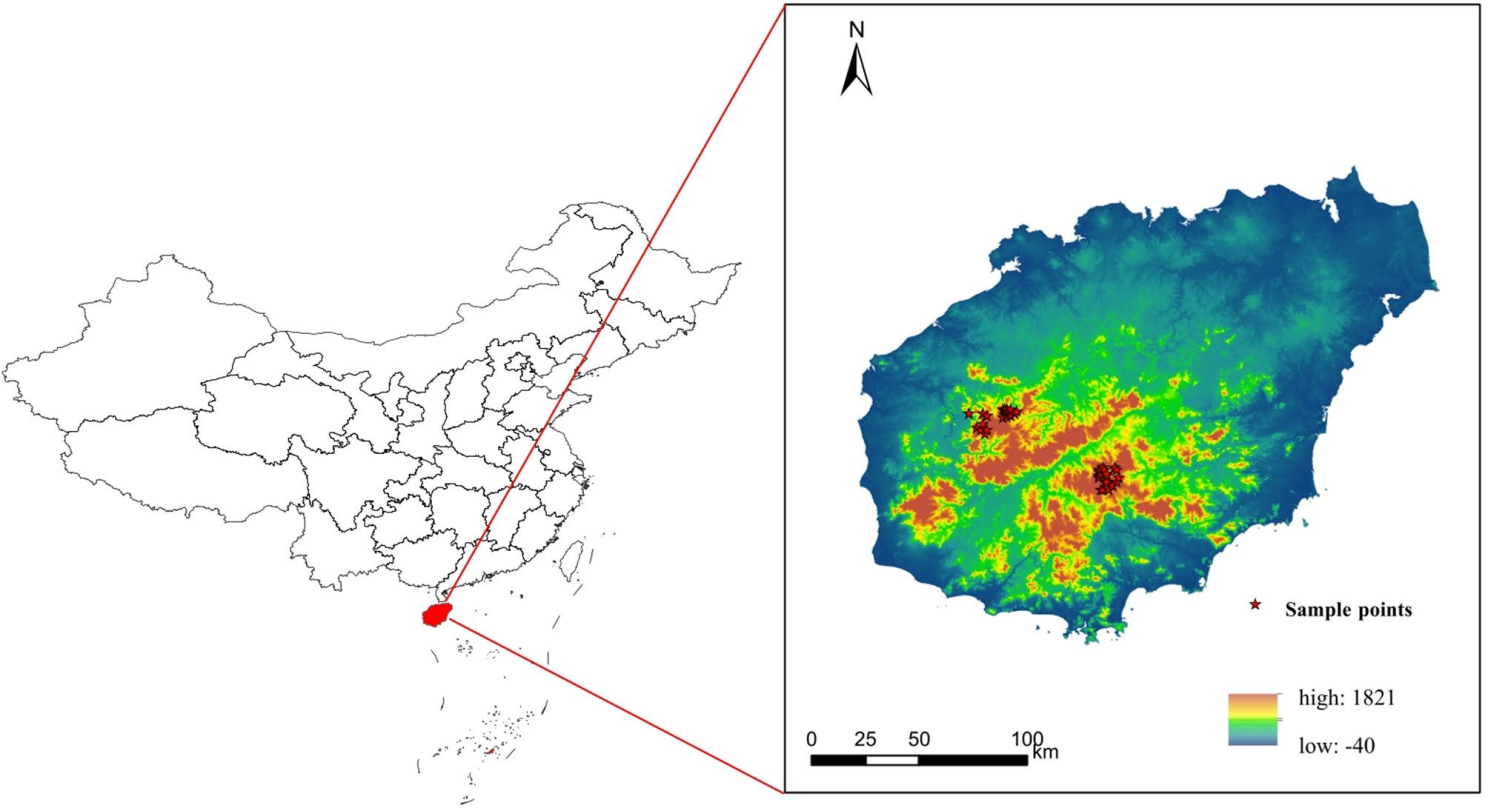
Map of distribution data of* Populus qiongdaoensis* in Hainan island. Note: Red five-pointed stars indicate recorded species sampling sites.

**Fig. 2:**
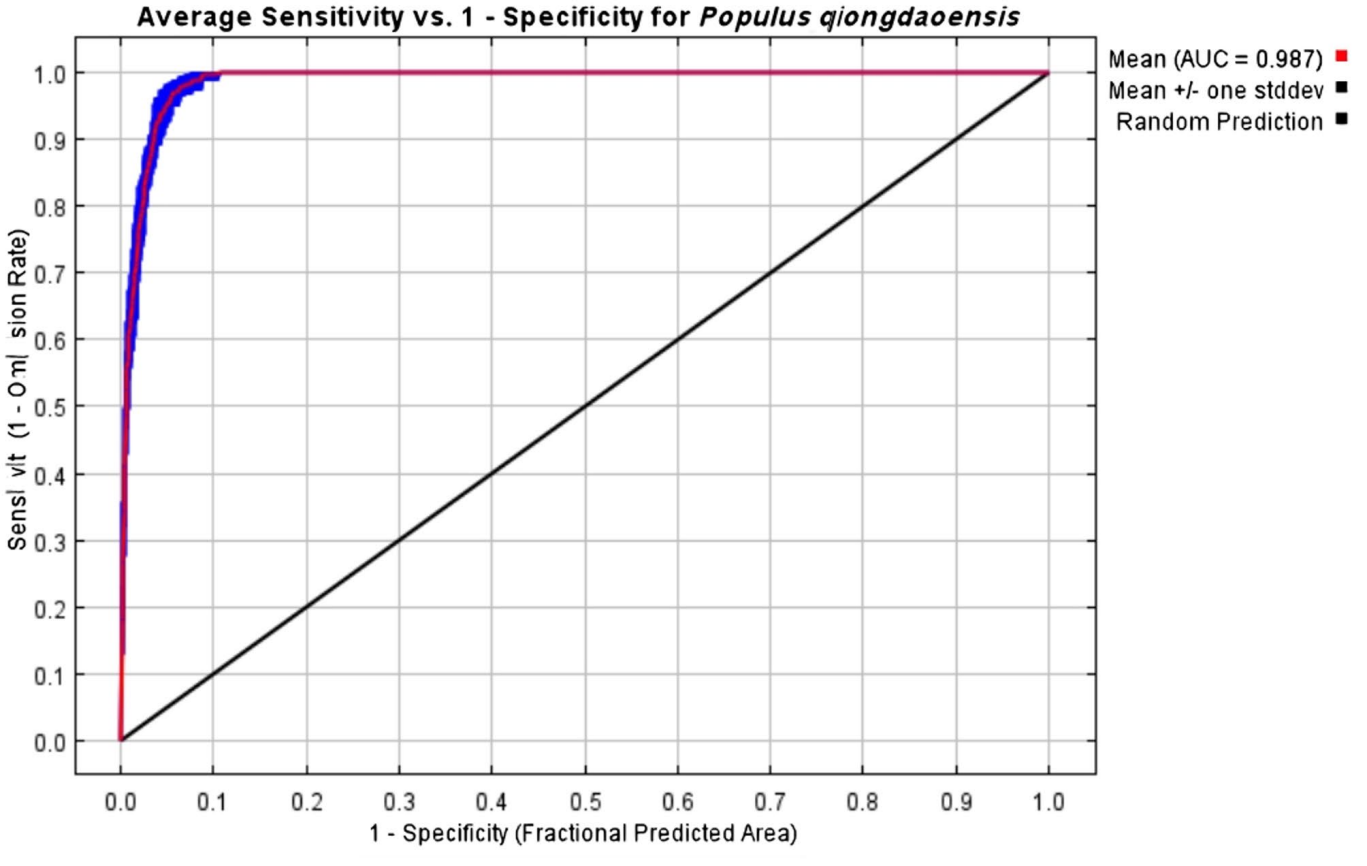
ROC curves and AUC averages for MaxEnt with 10 repeated runs.

We assessed the accuracy of the model for *P. qiongdaoensis* using a receiver operating characteristic (ROC) curve. The mean Area Under the Curve (AUC) value of the predictive distribution models constructed with MaxEnt software was 0.987 ± 0.003 (mean ± SD across 10 replicates; Fig. 1 for the sampling design, Fig. 2 for ROC curves), indicating excellent predictive performance. This high accuracy confirms the model’s reliability for identifying potential suitable habitats for *P. qiongdaoensis*.

### Contribution of environmental variables

**Fig. 3:**
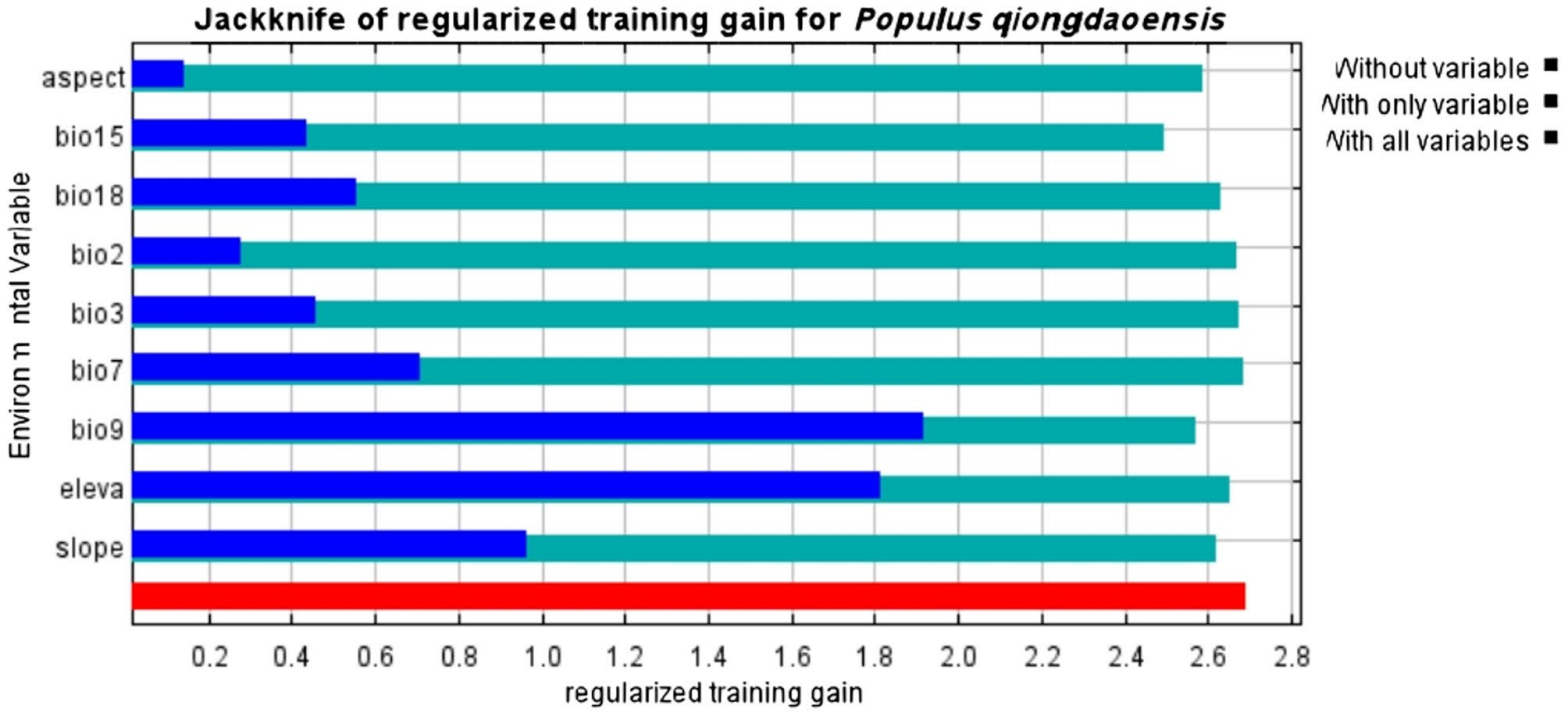
Jackknife test results showing the relative importance of environmental variables.

The importance of environmental factors to the modeling of adaptability of *P. qiongdaoensis* was simulated based on the jackknife method. The full set of environmental variables used in the model is listed in Table 1, and their contribution and permutation importance are shown in Table 2. The results showed that highest contribution rate of altitude (elev) was 63.7%, followed by the contribution rates of mean temperature of the driest quarter (bio9) and precipitation seasonality (bio15), which are 12.1% and 5.1%, respectively. These important variables have a great influence on the distribution of suitable habitats for *P. qiongdaoensis*, and contribute up to 80.9% of the cumulative contribution rate. It is particularly noteworthy that the mean temperature of the driest quarter (bio9) and precipitation seasonality (bio15) have ranking importance values of 48.6% and 11%, which are significantly higher than other variables. The results of the jackknife test showed that when considering a single environmental variable, the training gain of the model was significantly higher on mean temperature of the driest quarter (bio9) (Fig. 3), followed by elevation (elev) and slope (slope). When individual variables were ignored, the most significant decrease in model gains occurred on mean temperature of the driest quarter (bio9), indicating that it contained more effective information than other environmental variables, followed by elevation (elev) and slope (slope). According to the contribution rate and replacement importance of each environmental variable, elevation (elev), mean temperature of the driest quarter (bio9) and precipitation seasonality (bio15) are the main environmental variables affecting the distribution of populus in *P. qiongdaoensis*.

**Table 1:** Environmental factors involved in model construction.

Data type	Environment	Code name
Climatic factors	Annual mean temperature	biol
	Mean diurnal range	bio2
	Isothermality	bio3
	Temperature seasonality	bio4
	Max. temperature of the warmest month	bio5
	Min. temperature of the coldest month	bio6
	Temperature of annual range	bio7
	Mean temperature of the wettest quarter	bio8
	Mean temperature of the driest quarter	bio9
	Mean temperature of the warmest quarter	biol0
	Mean temperature of the coldest quarter	bioll
	Annual precipitation	bio12
	Precipitation of the wettest month	bio13
	Precipitation of the driest month	bio14
	Precipitation seasonality	bio15
	Precipitation of the wettest quarter	biol6
	Precipitation of the driest quarter	bio17
	Precipitation of the warmest quarter	biol8
	Precipitation of the coldest quarter	bio19
Terrain variables	Elevation	elev
	Aspect	aspect
	Slope	slope

**Table 2:** Contribution rate of environmental factors of *P. qiongdaoensis*.

Environmental variable	Description	Percent contribution (%)	Permutation importance (%)
eleva		63.7	9
bio9	Mean temperature of the driest quarter	12.1	48.6
bio15	Precipitation seasonality	5.1	11
bio2	Mean diurnal range	5	3.3
aspect		4.8	2.9
slope		4.2	1.7
bio18	Precipitation of the warmest quarter	4.2	7.7
bio3	Isothermality	0.6	8.7
bio7	Temperature annual range	0.3	7.1

**Table 3:** Areas of suitable habitats for *P. qiongdaoensis* in Hainan under different climate scenarios (km^2^). The table shows the area of poorly, moderately, and highly suitability classes, and the total suitable area for the baseline period, 2050s, and 2090s under four SSP scenarios.

Scenarios	Highly suitable habitats	Moderately suitable habitats	Poorly suitable habitats	Total suitable habitats
Current	517	913	1306	2736
2050s_SSP126	434	1151	1617	3202
2050s_SSP245	603	1101	1772	3476
2050s_SSP370	724	1162	1544	3430
2050s_SSP585	626	1097	1407	3130
2090s_SSP126	553	1019	1611	3183
2090s_SSP245	653	1151	1867	3671
2090s_SSP370	574	934	1678	3186
2090s_SSP585	643	1151	1582	3376

### Adaptation threshold based on response curve

**Fig. 4:**
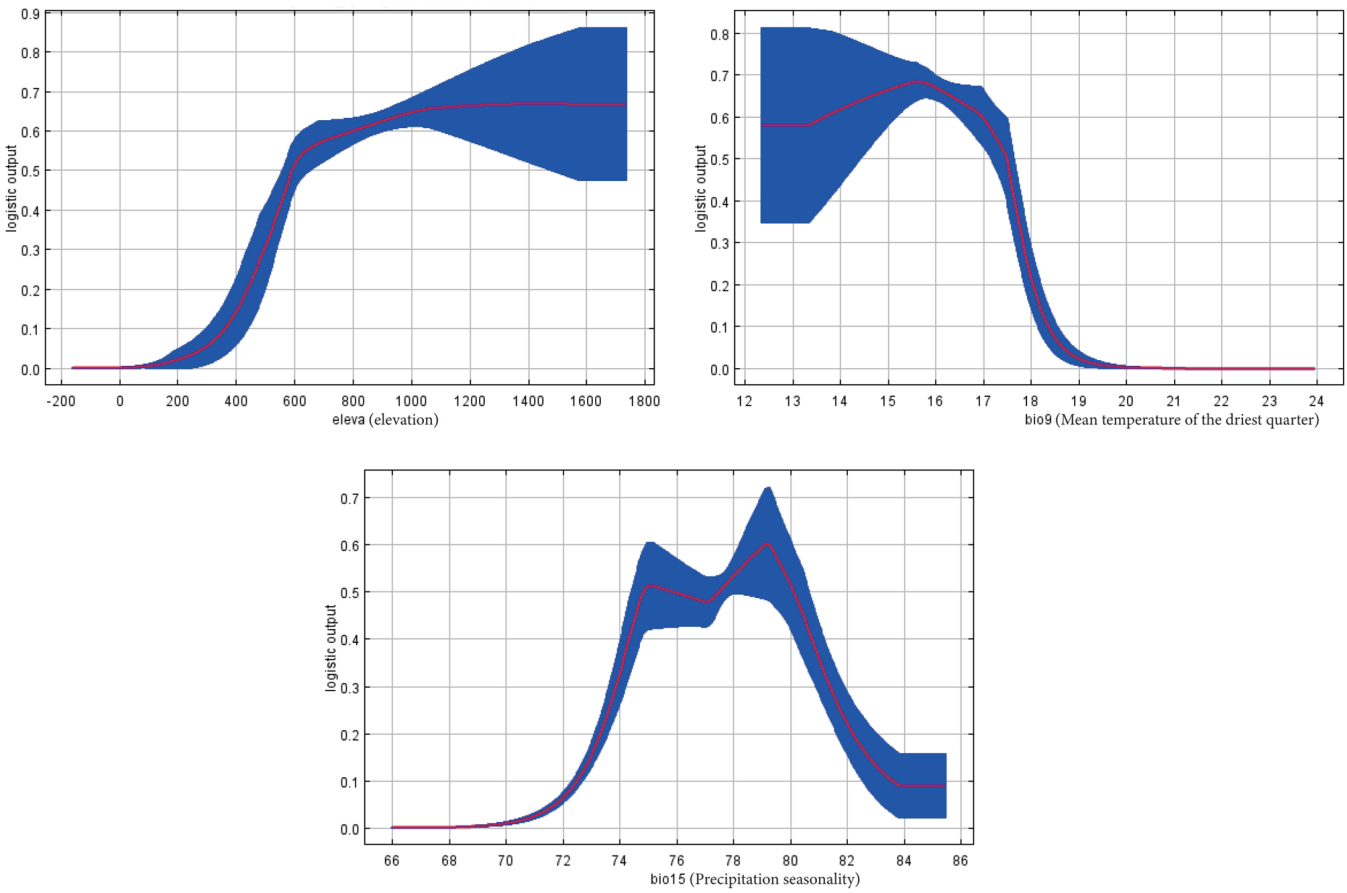
Response curves of the significant bioclimatic variables affecting the potential distribution of *P. qiongdaoensis*.

The Logistic coefficient is often used as the growth index of population and environmental factors. When the distribution probability *P* > 0.5, it is considered that the range of environmental factors is suitable for the growth and reproduction of *P. qiongdaoensis*. The results are shown in Fig. 4, in terms of altitude (elevation), the presence probability of *P. qiongdaoensis* increased rapidly with increasing altitude, and the most suitable range was 591.34–1633.61 m. Based on mean temperature of driest quarter (bio9), the probability of the presence of *P. qiongdaoensis* is greater than 0.5 when the temperature is between 12.48 °C and 17.58 °C, but when the temperature exceeds 17.58 °C, the probability of its presence decreases significantly. The most suitable range for precipitation seasonality (bio15) is 74.41–80.51 mm, with a maximum value of 79.13 mm.

### Potential distribution of *P. qiongdaoensis* under current climate

**Fig. 5:**
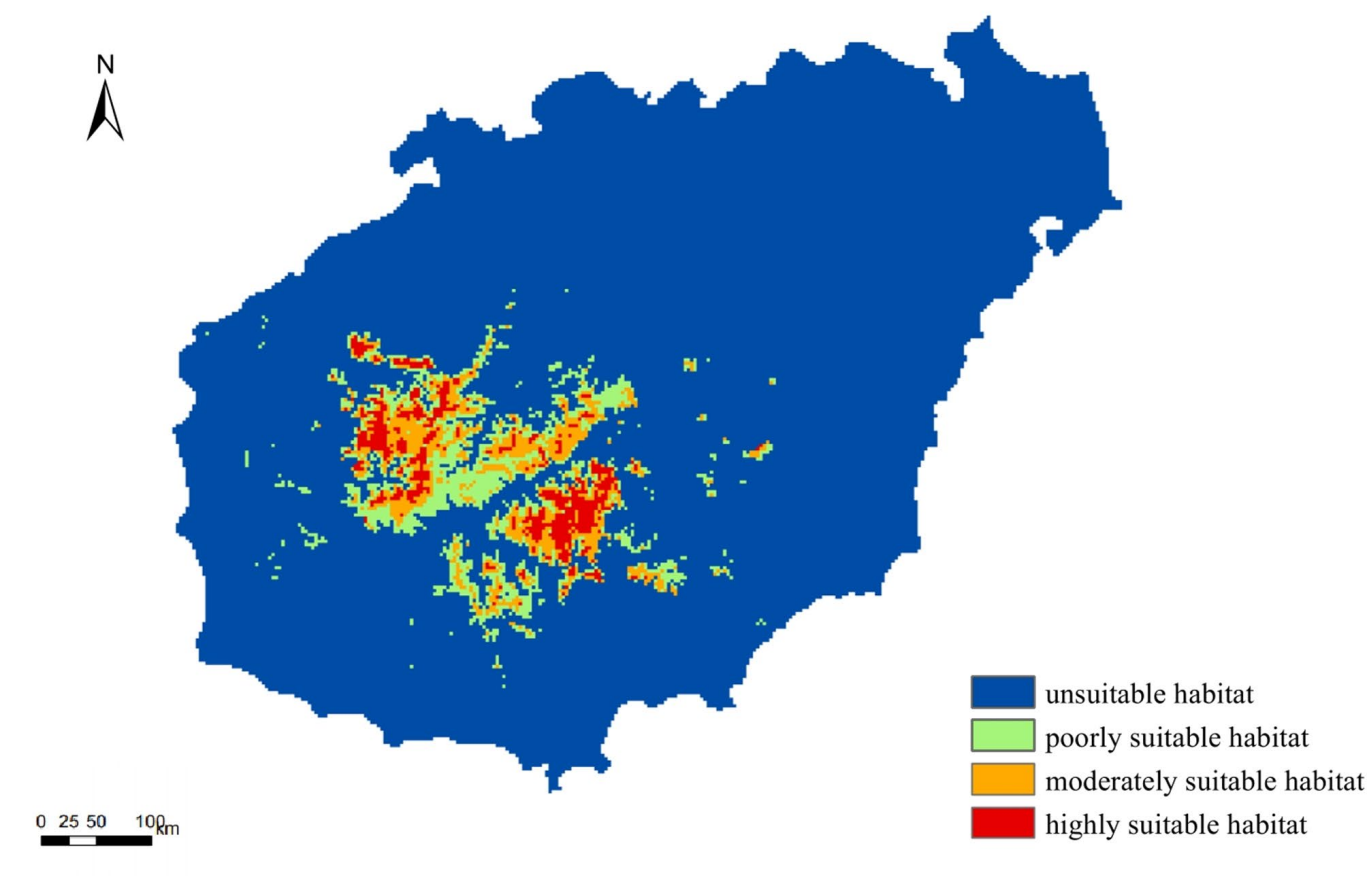
Suitable habitats for *P. qiongdaoensis* under current climate scenarios.

Under current climatic conditions, suitable habitats of *P. qiongdaoensis* are primarily concentrated in the central and southwestern mountainous regions of Hainan Island, particularly in Changjiang, Baisha, and Wuzhishan (Fig. 5). The total area of suitable habitat was estimated at 2736 km², accounting for 9.60% of the total land area of Hainan Island. Highly suitable habitats covered 517 km² (18.90% of the total suitable area) and were mainly distributed in the Bawangling and Wuzhishan regions. Moderately suitable habitats accounted for 913 km² (33.37%), while low-suitability habitats comprised 1306 km²(47.73%). Overall, the current distribution pattern indicates a strong association between suitable habitats and mountainous forested areas.

### Potential distribution of *P. qiongdaoensis* under future climate

**Fig. 6:**
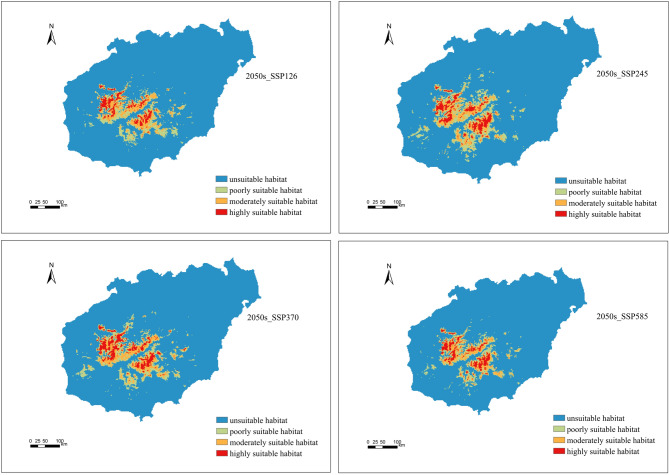
Suitable habitats of *P. qiongdaoensis* under four climatic scenarios in the 2050s.

**Fig. 7:**
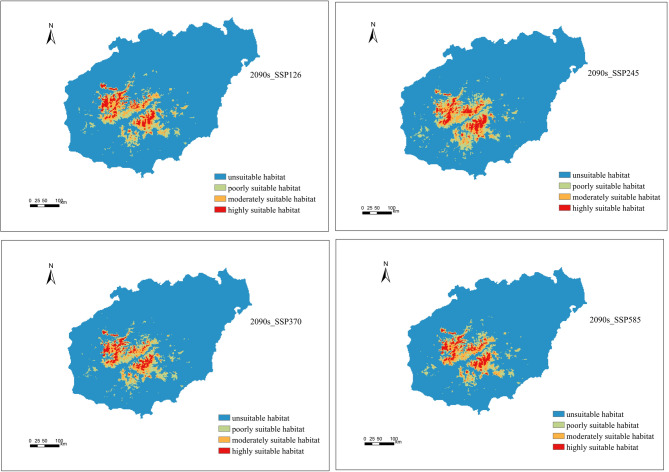
Suitable habitats of *P. qiongdaoensis* under four climatic scenarios in the 2090s.

The projected changes in suitable habitat areas under different climate scenarios are summarized in Table 3. The spatial extent of suitable habitat for *P. qiongdaoensis* varied markedly among suitability classes and emission scenarios (Figs. 6 and 7). Under current climatic conditions, highly suitable, moderately suitable, and poorly suitable habitats covered 517 km², 913 km², and 1306 km², respectively .

**Fig. 8:**
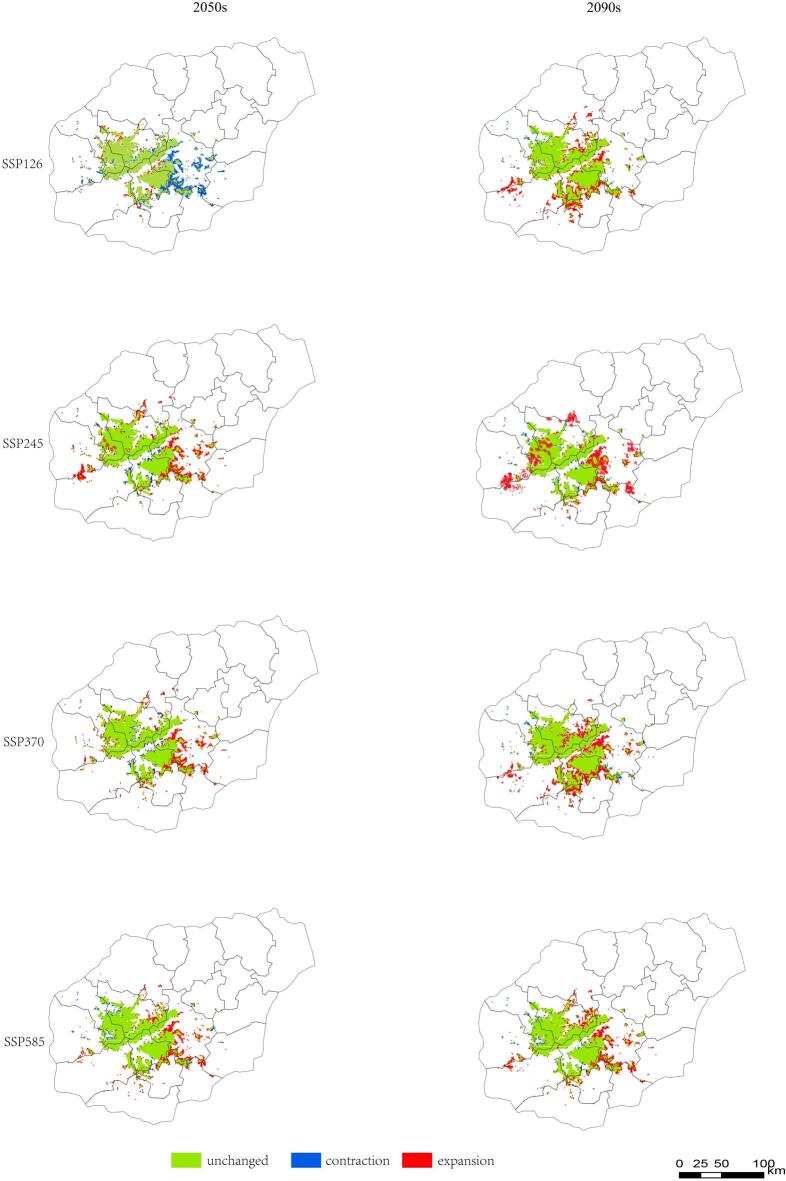
Changes of the suitable habitats of *P. qiongdaoensis* under different climate scenarios in 2050s and 2090s. The changes are categorized into three types: unchanged (areas remaining suitable under both current and future climates), expansion (areas becoming suitable in the future), contraction (areas becoming unsuitable in the future). The maps were generated by comparing the future projections from the MaxEnt model with the current potential distribution.

By the 2050s, projected habitat changes differed substantially among scenarios. Under the low-emission SSP126 scenario, the area of all suitability classes declined, with the largest reduction observed in poorly suitable habitat (− 22.97%). In contrast, SSP245, SSP370, and SSP585 projected overall increases in suitable habitat area (Fig. 8; Table 4). The largest expansion of highly suitable habitat occurred under SSP370 (+ 40.03%), whereas SSP245 produced the greatest increases in moderately suitable (+ 20.59%) and poorly suitable (+ 35.68%) habitats.

**Table 4:** Changes in the area of suitable habitat for *P. qiongdaoensis* under future climate scenarios (unit: km^2^). The table shows the projected gains, losses, and stable areas for total habitat and for each suitability class (poorly, moderate, highly) in the 2050s and 2090s across four SSP scenarios, compared to the current baseline.

Scenarios	Highly suitable habitats	Changes (%)	Moderately suitable habitats	Changes (%)	Poorly suitable habitats	Changes (%)
Current	517	0	913	0	1306	0
2050s_SSP126	434	-16.05	840	-7.99	1006	-22.97
2050s_SSP245	603	16.63	1101	20.59	1772	35.68
2050s_SSP370	724	40.03	1162	27.27	1544	18.22
2050s_SSP585	626	21.08	1097	20.15	1407	7.733
2090s_SSP126	553	6.96	1019	11.61	1611	23.35
2090s_SSP245	653	26.31	1151	26.06	1867	42.95
2090s_SSP370	574	11.02	934	2.3	1678	28.48
2090s_SSP585	643	24.37	1151	26.06	1582	21.13

**Fig. 9:**
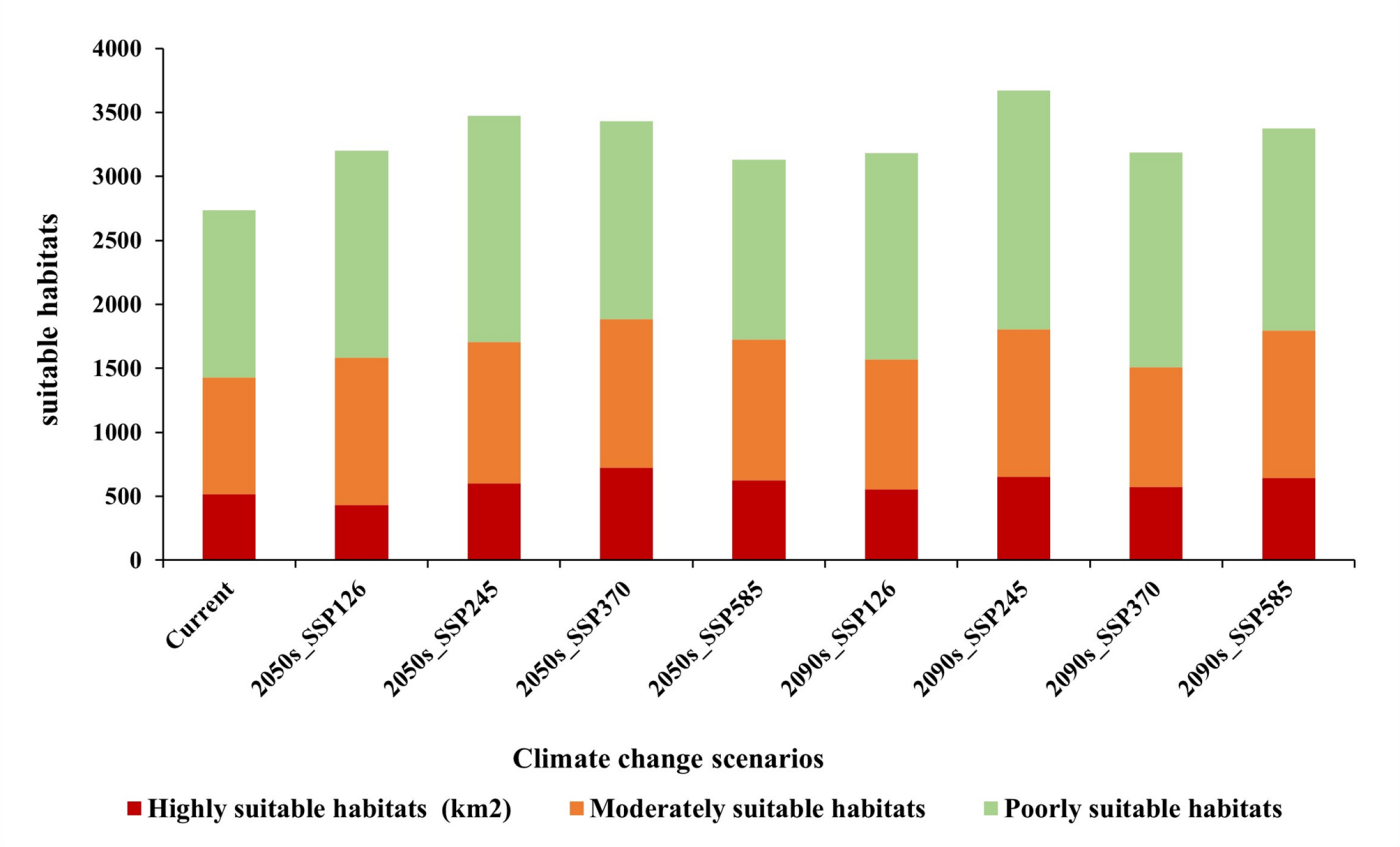
Area of potential suitable habitat for *P. qiongdaoensis* under current and future climates. The bars represent the total area of poorly, moderately, and highly suitability classes for the baseline period and future scenarios (2050s, 2090s; SSP126, 245, 370, 585), as projected by the MaxEnt model. Area is given in km^2^.

By the 2090s, habitat areas under SSP126 rebounded, resulting in net increases across all suitability classes, including a 23.35% increase in poorly suitable habitat relative to the current baseline. The SSP245 scenario showed the most extensive expansion overall, with increases of 26.31%, 26.06%, and 42.95% in highly suitable, moderately suitable, and poorly suitable habitats, respectively. Under SSP370, the expansion rates of highly and moderately suitable habitats in the 2090s were lower than those projected for the 2050s, while SSP585 continued to show consistent increases exceeding 20% for both classes (Fig. 9).

### Spatial pattern changes in suitable habitats under future climate scenarios

**Fig. 10:**
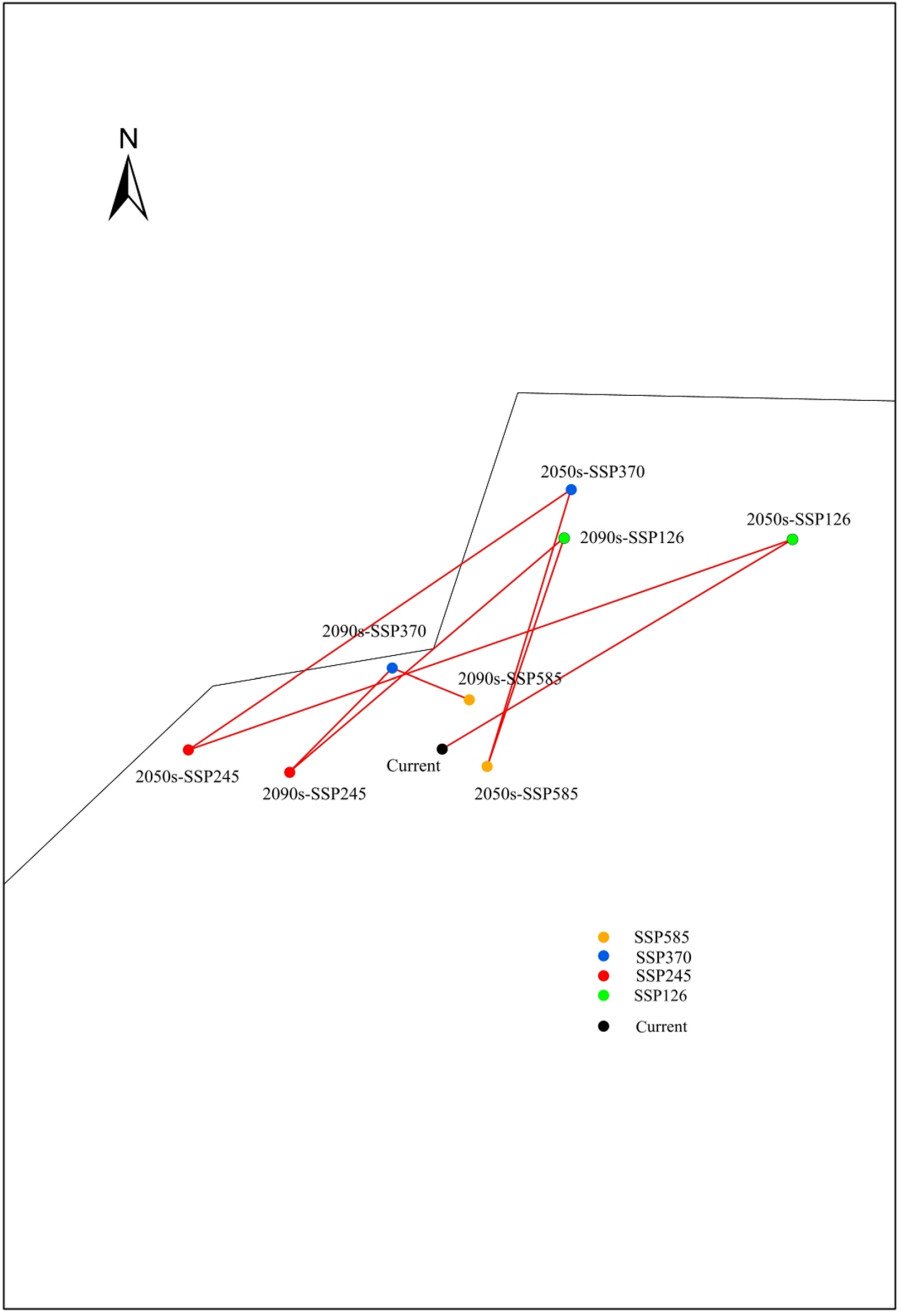
Change of centroid in suitable area of *P. qiongdaoensis* under current and future climatic conditions.

An analysis of the centroid shifts and migration patterns of suitable habitats under different climate scenarios was conducted. The results indicate that under the current climate scenario, the distribution centroid of the suitable habitat for *P. qiongdaoensis* is located in the northwestern part of Wuzhishan City, with coordinates 18.981368°N, 109.457562°E. Under the four future climate scenarios with varying CO₂ emission levels, the centroid is projected to shift to different extents (Fig. 10).

Under the SSP126 climate scenario, the distribution centroid of *P. qiongdaoensis* is projected to shift northeastward by 2050, reaching coordinates 19.021312°N, 109.517552°E, with a displacement of 7.54 km (Table 5). By 2090, it is expected to move further westward to 19.011494°N, 109.474953°E, corresponding to a shift of 3.83 km. Under the SSP245 scenario, the centroid will shift westward by 2050 to 18.981274°N, 109.421338°E (displacement: 3.81 km), followed by an eastward movement by 2090 to 18.97807°N, 109.435784°E (displacement: 2.32 km). In the SSP370 scenario, the centroid shifts northeastward by 2050 to 19.018395°N, 109.475952°E (displacement: 4.55 km), then shifts southwestward by 2090 to 18.992932°N, 109.450415°E (displacement: 1.49 km). Under the SSP585 scenario, the centroid first moves eastward by 2050 to 18.978906°N, 109.463957°E (displacement: 0.73 km) and subsequently shifts northward by 2090 to 18.988432°N, 109.461413°E (displacement: 0.88 km). Overall, the east-west movement of the distribution center is not pronounced, whereas a consistent northward shift is observed. Under future climate conditions, the distribution centroid of suitable habitat for *P. qiongdaoensis* remains located within the territory of Wuzhishan City.

**Table 5:** Centroid coordinates and migration distances of suitable habitat for *P. qiongdaoensis* under current and future climate scenarios. The migration distance from the baseline centroid is provided in Kilometers (km).

Period	Longitude	Latitude	Distance/km
Current	109.457562°E	18.981368°N	
2050s_SSP126	109.517552°E	19.021312°N	7.54
2090s_SSP126	109.474953°E	19.011494°N	3.83
2050s_SSP245	109.421338°E	18.981274°N	3.81
2090s_SSP245	109.435784°E	18.97807°N	2.32
2050s_SSP370	109.475952°E	19.018395°N	4.55
2090s_SSP370	109.450415°E	18.992932°N	1.49
2050s_SSP585	109.463957°E	18.978906°N	0.73
2090s_SSP585	109.461413°E	18.988432°N	0.88

## Discussion

This study employed the MaxEnt model integrated with ArcGIS to successfully analyze the distribution of *P. qiongdaoensis*. The model demonstrated high accuracy, with all AUC values exceeding 0.9. The simulated highly suitable habitats under current climatic conditions closely aligned with the actual known distribution points of the species, further validating the reliability of the modeling results^27–29^.

The distribution of species is influenced by a variety of factors, with different species requiring distinct living environments and being constrained by different governing factors. Environmental variables often serve as key parameters determining biological growth, development, and geographical distribution^30^. Our MaxEnt model simulations identify elevation (elev), mean temperature of the driest quarter (bio9), and precipitation seasonality (bio15) as the dominant variables shaping the potential distribution of *P. qiongdaoensis*^31,32^. This is consistent with the unique montane tropical climate of Hainan Island and the known ecological habits of this species, which likely depends on cool, moist conditions found at higher elevations. Jackknife tests show that elevation had the highest training gain when used alone, while bio9 and bio15 had the highest permutation importance in the full model. This reflects a hierarchical control of distribution: elevation defines the broad montane zone, acting as a proxy for temperature, moisture, and solar radiation, whereas bio9 and bio15 impose specific climatic constraints within this zone. Bio9 likely sets thermal limits during the driest quarter, restricting areas that are too cold or too warm, while bio15 indicates dependence on a stable precipitation regime, with extremes increasing drought or disrupting phenological cues^33,34^.

Overall, these results suggest a two-tiered environmental filtering: elevation delineates the suitable montane habitat, and bio9 and bio15 refine its distribution through physiological thresholds. This interpretation resolves the apparent differences between Jackknife and permutation importance metrics and highlights the need to consider both topography and climate when predicting climate change impacts on the species.

The habitat suitability of *P. qiongdaoensis* exhibits distinct elevational zonation based on response curve analysis: at low elevations (< 400 m), suitability is minimal due to intense human disturbance, high temperature and humidity, and poor soil drainage that restricts root respiration. Suitability increases rapidly between 400 and 600 m, reaching a consistently high plateau within the 600–1000 m range, where temperature, moisture, topography, and habitat heterogeneity optimally support growth. Above 1200 m, suitability declines, likely as a result of low winter temperatures and shortened growing seasons that fail to meet the cumulative temperature requirements of this temperate-origin species. Moreover, *P. qiongdaoensis* shows a narrow adaptive range to the mean temperature of the driest quarter (bio9, 13–17 °C). Temperatures exceeding 17 °C cause a sharp decline in suitability due to elevated transpiration and metabolic stress, while values below 13 °C impair growth through tissue frost damage and reduced photosynthetic efficiency^35^. The species also displays strong dependence on precipitation seasonality (bio15), with an optimal range of 74–80 corresponding to the island’s monsoonal climate-characterized by dry winters and wet summers-which balances water availability during growth and moderate drought in the dry season^36^. Values above 80 exacerbate flooding and extreme drought, whereas values below 74 increase pathogen risk due to persistently high humidity. These response patterns reflect *P. qiongdaoensis*’ adaptation to the topoclimatic heterogeneity of Hainan Island and provide critical ecological parameters for its conservation and reintroduction.

Under current climatic conditions, *P. qiongdaoensis* is largely confined to the central and southwestern mountainous regions of Hainan Island, particularly Changjiang, Baisha, and Wuzhishan. Highly suitable habitats are limited, mainly concentrated in Bawangling and Wuzhishan, and account for less than 20% of total suitable area. Moderately and poorly suitable habitats dominate the remaining landscape, reflecting a strong association between the species’s distribution and forested montane areas. This pattern highlights the species’s narrow ecological niche and its dependence on relatively intact high-elevation habitats, where temperature, moisture, and topography collectively support optimal growth and survival^37,38^.

However, increases in total suitable area do not necessarily translate into improved habitat quality or demographic stability, as the proportion of highly suitable habitat remains limited relative to the total landscape. This “expansion-contraction dichotomy” underscores that climate change may simultaneously create new suitable areas while fragmenting existing high-quality habitats, potentially challenging species survival^39^.

Future climate projections indicate notable shifts in both the extent and spatial configuration of suitable habitats. By the 2050s, low-emission scenarios (SSP126) predict contractions across all suitability classes, particularly in marginal habitats, whereas moderate to high-emission scenarios (SSP245, SSP370, SSP585) generally forecast expansion, especially for highly suitable habitats under SSP370. By the 2090s, SSP245 shows the most extensive overall expansion, whereas SSP370 and SSP585 continue to exhibit increases, albeit with some temporal variation. These findings suggest that climate warming and elevated CO₂ concentrations may render additional areas suitable, particularly at higher elevations or in northern regions of the island^40,41^. However, increases in total suitable area do not necessarily translate into improved habitat quality or demographic stability, as the proportion of highly suitable habitat remains limited relative to the total landscape. This “expansion-contraction dichotomy” underscores that climate change may simultaneously create new suitable areas while fragmenting existing high-quality habitats, potentially challenging species survival^42^.

An interesting pattern emerges under SSP126, where highly suitable habitats are projected to decline by the 2050s, in contrast to the expansions observed under moderate and high-emission scenarios^43^. This apparent anomaly likely reflects the non-linear response of this high-elevation specialist to thermal and hydric changes within its narrow ecological niche^44^. Modest warming under SSP126 may shift core habitat microclimates slightly above or below physiological optima, reducing suitability without creating new favorable areas^45^. In contrast, larger temperature increases and altered precipitation patterns under SSP245, SSP370, and SSP585 open additional areas at higher elevations or in northern regions, offsetting losses and increasing high-suitability zones. These findings illustrate that slight warming may temporarily degrade core habitats, whereas more substantial warming can generate compensatory suitable areas elsewhere^43^.

By analyzing the distribution centroids, we further elucidated the species’s response to climate change. Across all simulated scenarios, the centroid exhibited a consistent northwestward and northward shift, ultimately moving northward, although east-west displacements were relatively small and the centroid remained within Wuzhishan City^46^. This indicates the continued persistence of core montane refugia. The modest centroid displacements (ranging from less than 1 km to approximately 7.5 km) suggest that *P. qiongdaoensis* may require only limited dispersal to track climate-driven shifts in its ecological niche over the short term. Nevertheless, natural dispersal could be constrained by habitat fragmentation and human activities, highlighting the potential need for assisted migration or enhanced habitat connectivity^47^.

Several limitations should be acknowledged. First, MaxEnt assumes equilibrium between species distribution and current environmental conditions and does not explicitly account for dispersal limitations, biotic interactions, or demographic processes^48^. Second, land-use change and deforestation-significant factors on Hainan Island-were not incorporated into the model, potentially leading to overestimation of suitable habitats^49^. Third, future projections were based on a single GCM, which may not capture the full range of climatic uncertainty^50^. Future studies should integrate land-use/land-cover data, dispersal constraints, and ensemble modeling approaches using multiple GCMs to improve prediction robustness.

Our findings provide important guidance for the conservation of *P. qiongdaoensis*. Priority should be given to protecting and restoring habitats in Bawangling and Wuzhishan, which are projected to remain highly suitable under future climates. Assisted migration and habitat connectivity enhancement may help facilitate range shifts^51^. Moreover, integrating genetic studies on heat and drought tolerance with spatial modeling could improve conservation planning and ensure long-term population resilience^52^. Future research should combine field validation, genetic adaptation analyses, and dynamic population models to refine predictions and support evidence-based conservation strategies under ongoing climate change.

## Conclusions

This study highlights the vulnerability and ecological specificity of *Populus qiongdaoensis*, a tropical endemic tree restricted to Hainan Island. Elevation, mean temperature of the driest quarter, and precipitation seasonality were identified as key factors shaping its distribution, emphasizing the importance of montane refugia for its persistence. Projected climate change may alter the extent and configuration of suitable habitats, potentially creating new areas in northern and higher-elevation regions, while the proportion of highly suitable habitats remains limited.

These insights provide a theoretical basis for targeted conservation actions. Priority should be given to monitoring and protecting core populations in Wuzhishan, which are likely to remain climatically stable. Additionally, assisted planting or habitat restoration in northeastern regions may facilitate future range shifts and enhance species resilience. Integrating ecological monitoring with management interventions will be crucial to safeguard *P. qiongdaoensis* under ongoing and future climate change, offering a practical framework for conserving other tropical island endemics facing similar environmental pressures.

## Materials and methods

### Species occurrence data collection and processing

Occurrence records of *P. qiongdaoensis* on Hainan Island were compiled from multiple sources, including the Global Biodiversity Information Facility (GBIF; https://www.gbif.org), the Flora of China database (https://www.iplant.cn), and field surveys conducted by the authors. A total of 76 initial records with geographic coordinates were obtained. To mitigate potential spatial sampling bias and autocorrelation arising from clustered records, we performed spatial filtering using ENMTools^53^. This process involved removing duplicate and spatially redundant points within the same raster cell, resulting in 42 spatially independent and valid occurrence records for subsequent analysis (Fig. 2). These filtered records were saved in CSV format for use in the MaxEnt model construction.

### Environment variable filtering

To comprehensively analyze the relationship between the distribution of *P. qiongdaoensis* in Hainan Island and environmental factors, 19 bioclimatic variables and 3 terrain variables were used with a spatial resolution of 30 arc-seconds (~ 1 km) (Table 1). The 19 climate variables used in this study were all sourced from the WorldClim database (https://worldclim.org/ (accessed on 21 December 2023)), encompassing both current climate data (1970–2000) and future climate projections 2050s (2041 ~ 2060) and 2090s (2081 ~ 2100). The future climate data originated from the Moderate Resolution National Climate Center (Beijing) Climate System Model (BCC-CSM2-MR) within the Coupled Model Intercomparison Project Phase 6 (CMIP6). This model is recognized as being particularly suitable for simulating climate conditions in China^54^. Future climate change scenarios included four Shared Socioeconomic Pathways (SSPs): low radiative forcing (SSP126), medium radiative forcing (SSP245), medium-to-high radiative forcing (SSP370), and high radiative forcing (SSP585)^55^. Terrain data were derived from a 1-km resolution Digital Elevation Model (DEM) downloaded from the Geospatial Data Cloud platform (https://www.gscloud.cn/). Using ArcGIS 10.8, three topographic layers were generated from this DEM: Elevation, Slope, and Aspect^19^.

### Environmental factor preprocessing

To avoid model overfitting due to multicollinearity, we performed a two-step variable selection. First, we calculated pairwise Pearson correlation coefficients for all 22 variables within the study extent using ENMTools. For any pair with |r| > 0.8 ^56,57^, we retained the variable considered more biologically relevant for the species based on prior ecological knowledge. Second, we ran a preliminary MaxEnt model with the remaining variables and used the Jackknife test of variable importance to eliminate variables with negligible permutation importance (< 1%). This procedure resulted in a final set of 8 bioclimatic and 3 topographic variables for modeling (Table 1).

### Construction and accuracy optimization of maxent models

MaxEnt models were constructed using the 11 selected environmental variables and the 42 filtered occurrence records. Models were run using a cross-validation approach with 10 replicates, in which 75% of the occurrence records were used for training and 25% for testing in each run^58,59^. The random seed option was enabled to ensure independent partitions among replicates. Feature classes and regularization multipliers were set to automatic (default) values, which have been shown to provide robust performance for species with limited occurrence records. Response curves and Jackknife tests were used to evaluate species-environment relationships. Model outputs were generated in logistic format.

Model performance was evaluated using the area under the receiver operating characteristic curve (AUC). Mean AUC values across replicates were used to assess overall model accuracy, with values closer to 1 indicating better discriminatory ability^60^.

### Habitat suitability classification and change analysis

Continuous habitat suitability outputs generated by the MaxEnt model were converted into raster layers and processed in ArcGIS version 10.8. Habitat suitability was reclassified using the natural breaks (Jenks) classification method, which minimizes within-class variance and is commonly applied to continuous suitability outputs in species distribution modeling studies. Based on this method, habitat suitability was divided into four categories: unsuitable (0-0.08), low suitability (0.09–0.26), moderate suitability (0.26–0.46), and high suitability (0.47–0.92).

To quantify spatial changes in habitat suitability under different climate scenarios, the area and proportion of each suitability category were calculated from raster attribute tables. Changes in suitable habitat, including expansion, contraction, and stable areas, were analyzed using SDMTools. In addition, shifts in the centroid (mean center) of suitable habitat were calculated to evaluate the direction and magnitude of potential distributional changes of *P. qiongdaoensis* under future climate scenarios^19,61^.

### Software and tools

All analyses were conducted using the following software to ensure reproducibility: MaxEnt (v3.4.4), Java Runtime Environment (v8), ArcGIS (v10.8.2, ESRI), and SDMtoolbox (v2.4). Statistical screening was performed in R (v4.3.1) with the ENMTools package. All specific parameters and settings used in the analyses are detailed in the respective sections above.

## Data Availability

The data supporting the findings of this study are included in the article, and furtherinquiries can be directed to the corresponding author.
